# Retrospective clinical study of mandible fractures

**DOI:** 10.1186/s40902-022-00365-3

**Published:** 2022-11-02

**Authors:** Seungjin Cha, Gaeun Park, Baek-Soo Lee, Yong-Dae Kwon, Byung-Joon Choi, Jung-Woo Lee, Junho Jung, Jooyoung Ohe

**Affiliations:** Department of Oral and Maxillofacial Surgery, School of Dentistry, Graduate School, Kyung Hee University, Seoul, Republic of Korea

**Keywords:** Mandibular fractures, Trauma, Jaw fractures

## Abstract

**Background:**

As society becomes more complex, the incidence of mandibular fractures is increasing. This study aimed to analyze the incidence and type and identify etiological factors of mandibular fractures to use them in future treatments.

**Material and methods:**

Data were collected from 224 patients who visited the department of oral and maxillofacial surgery at the Kyung Hee Medical Center dental hospital during a 6-year period (2016 to 2021). A logistic regression model was used for data analysis.

**Results:**

In a total of 224 patients, 362 fractures were appeared. The average age of the patients was 34.1 years, with the highest incidence in the 20s. And the ratio between male and female was 4.09:1. Symphysis fractures were the most prevalent of all patients (52.7%), followed by unilateral condyle (37.1%), angle (36.2%), bilateral condyle (9.4%), body (8%), and coronoid (2.2%). The most common cause of fracture was daily-life activity (57.6%), followed by violence (30.4%), traffic accidents (8.5%), and syncope (3.6%). Patients with symphysis fracture were at low risk (*OR* < 1) of angle, body, and unilateral condyle fractures. Similarly, patients with unilateral fracture were at low risk (*OR* < 1) of symphysis, angle, body, and others site fractures. In contrast, patient with bilateral condyle fracture were at high risk (*OR* > 1) of coronoid fractures. And younger patients were high risk of mandibular angle fractures.

**Conclusion:**

Through this study, it was confirmed that etiological factors of mandibular fractures were like those of previous studies.

## Background

As society gradually became a developed country through industrialization and urbanization, it was observed that modern people’s opportunities for sports and leisure activities increased. These social changes not only affect an individual’s lifestyle but also cause an increase in injuries. Due to the protruding anatomical structure of the maxillofacial region, it is prone to injuries and fractures. The mandible is the only movable bone among the maxillofacial bones, and the frequency of fractures is higher than that of other parts of the body [1, 2]. Factors that affect the location of a mandibles fracture include internal factors such as age, state of health and other parts of the fracture, and external factors such as the direction and size of the external forces applied. Several studies have analyzed the mechanisms by which mandible fractures occur, but external factors are broadly variable. A comprehensive understanding of the many factors that affect location of mandibular fracture is needed, and we focused on internal factors. This article analyzes the mechanics of mandible fracture from an internal point of view. Therefore, this study aims to identify and distinguish these internal factors using a binomial logistic regression analysis. Through this, we intend to check the tendency of mandibular fractures and reference to the treatment of patients in the future.

According to domestic and foreign previous studies on mandibular fractures, the causes of fractures are classified into falls, violence, and traffic accidents [3], and fracture sites are classified into the symphysis, mandible angle, mandibular body, mandibular condyle, and coronoid process [1, 4, 5]. It is thought that etiological factors such as the cause of the fracture, the location of the fracture, the patient’s age, health status, and the presence or absence of smoking and drinking have an influence on the pattern and distribution of fractures [6, 7]. In this study, the etiological factors such as age, gender, cause of fracture, fracture location, and patient's health status were investigated for patients who visited the department of oral and maxillofacial surgery at the Kyung Hee Medical Center Dental Hospital from 2016 to 2021 based on analysis.

## Materials and methods

In this study, the etiological factors such as age, gender, cause of fracture, fracture location, and patient’s health status were investigated for 224 patients who visited the department of oral and maxillofacial surgery at the Kyung Hee Medical Center Dental Hospital from 2016 to 2021 with maxillofacial fracture, based on analysis. The data were classified into age, sex, maxillofacial fracture type, cause of trauma, smoking, and drinking alcohol, which collected and standardized by an investigator based on the case histories, medical records of the patients, and clinical and radiographic examinations. Mandibular fractures were classified as symphysis, angle, body, condyle (unilateral or bilateral), coronoid fractures, and maxillofacial fractures in other sites. Cause of trauma was classified as daily-life activity (such as falling and collision), violence, traffic accident, and syncope. We have followed the ethical guidelines of the Declaration of Helsinki in this study. All protocols were approved by the Institutional Review Board (IRB) of Kyung Hee University. The data were analyzed through descriptive statistical analysis and binomial logistic regression analysis. Through this analysis, this study aims to identify and distinguish the internal factors of mandibles fractures. The statistical analysis was performed using SPSS (SPSS version 25.0, Chicago, IL, USA). *p*-value less than 0.05 was considered statistically significant.

## Results

### Percentage of fracture distribution by gender and age

**Fig. 1:**
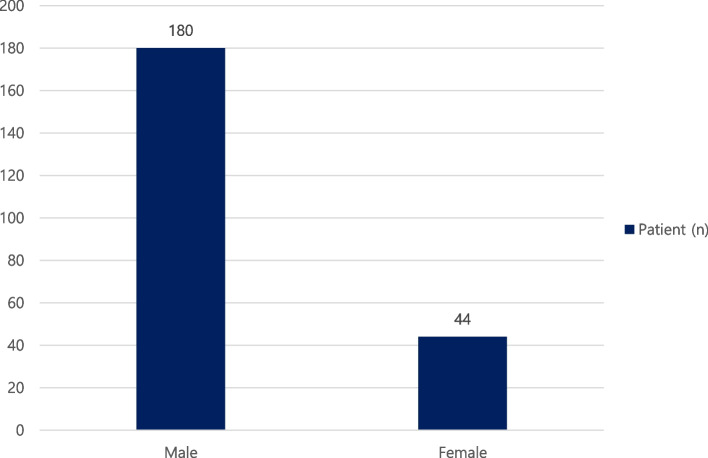
Distribution of gender

Of the total 224 patients, 180 patients (80.3%) were male, and 44 patients (19.7%) were female (Fig. 1). The ratio between males’ patients and females’ patients was 4.09:1. The ages of patients ranged from 9 to 92 years old, with an average age of 34.1. The patients were divided into 10-year increments according to age. The largest number of patients was in their 20s with 83 patients (37.1%). This was followed by teens (39 patients, 17.4%), 40s (36 patients, 16.1%), 50s (25 patients, 11.2%), 30s (22 patients, 9.8%), and over 60s (18 patients, 8.0%) (Table 1).

**Table 1:** Distribution of age

Age (year)	Patient (*n*)
10s	39
20s	83
30s	22
40s	36
50s	25
Over 60s	18
Total	224

### *Frequency of occurrence due to fractures*

**Fig. 2:**
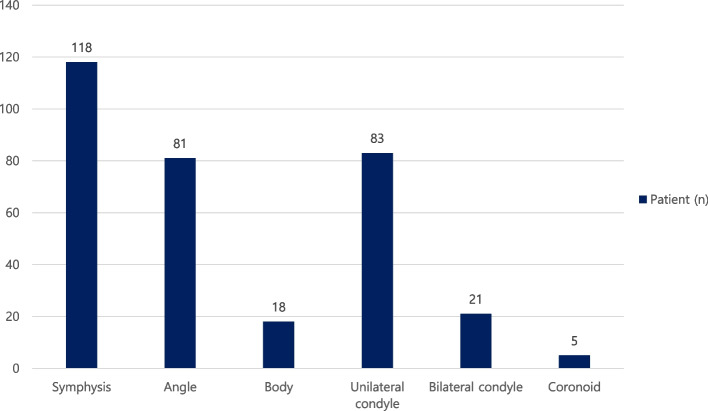
Distribution of fracture site

This study included 362 fractures in 224 patients. The average number of fracture lines per patient was 1.61. Among the patients, there were 115 patients (51.3%) with single fracture line, 102 patients (45.5%) with two fracture lines, and 7 patients (3.2%) with three or more fracture lines (Table 2). Symphysis fractures were the most among all patients (118, 52.7%), followed by 83 patients (37.1%) with unilateral condyle fractures, 81 patients (36.2%) with angle fractures, 21 patients (9.4%) with bilateral condyle fractures, 18 patients (8.0%) with body fractures, and 5 patients (2.2%) with coronoid fractures (Fig. 2). In a total of 109 patients with multiple fractures, there were 48 patients (44%) with symphysis and unilateral condyle fractures, followed by 26 patients (23.9%) with symphysis and angle fractures. Additionally, there were 15 patients (13.8%) with symphysis and bilateral condyle fractures and 3 patients (2.8%) with angle and body fractures (Table 3).

**Table 2:** Distribution of number of fracture lines

Fracture line	Patient (*n*)
1	115
2	102
More than 3	7
Total	224

**Table 3:** Distribution patterns of multiple fracture

Fracture site	Patient (*n*)
Symphysis & angle	26
Symphysis & body	1
Symphysis & unilateral condyle	48
Symphysis & bilateral condyle	15
Symphysis & coronoid process	1
Symphysis & others	1
Symphysis, body, bilateral condyle, & coronoid process	1
Symphysis, unilateral condyle, & others	2
Symphysis, unilateral condyle, & coronoid process	1
Symphysis, bilateral condyle, & coronoid process	1
Symphysis, bilateral condyle, & others	1
Angle & body	3
Angle & unilateral condyle	2
Angle, unilateral condyle, & coronoid process	1
Body & unilateral condyle	2
Body & bilateral condyle	1
Unilateral condyle & others	1
Bilateral condyle & others	1
Total	109

### *Cause of fractures*

**Fig. 3:**
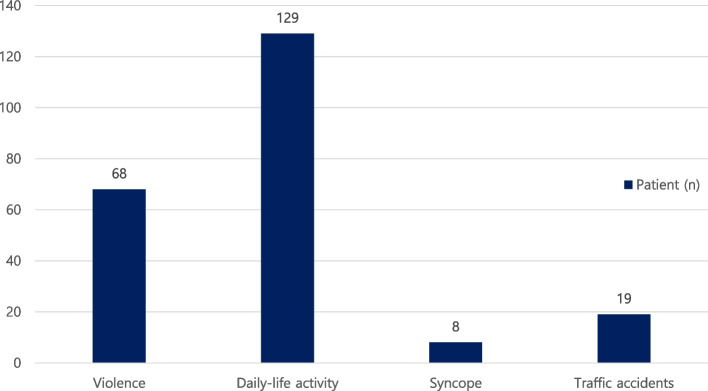
Distribution of fracture causes

Of the total 224 patients, 129 patients (57.6%) were fractured by daily-life activity, including falling and collision. Sixty-eight patients (30.4%) were fractured by violence, followed by 19 patients (8.5%) with traffic accidents, and 8 patients (3.6%) with syncope (Fig. 3). The causes of fractures were divided into before and after the COVID-19 pandemic. Of the total 153 fracture patients from 2016 to 2019, 79 patients (51.6%) were caused by daily-life activity, 55 patients (35.9%) were caused by violence, 14 patients (9.2%) were caused by traffic accidents, and 5 patients (3.3%) were caused by syncope. From 2020 to 2021, after the Covid-19 pandemic, there were a total of 71 fracture patients, 50 (70.4%) patients were caused by daily-life activity, 13 patients (18.3%) with violence, 5 patients (7%) with traffic accidents, and 3 patients (4.2%) with syncope.

### Factor analysis affecting the position of mandible fractures through binomial logistic regression analysis

According to logistic regression analysis of patients with symphysis fractures, there has been a significant correlation with mandibular angle, body, and unilateral condyle fractures (*p*-value < 0.05). Patients with symphysis fractures were at low risk (*OR* < 1) of angle, body, and unilateral condyle fractures (Table 4). In the case of mandibular angle fractures, logistic regression showed a significant correlation between ages (*p*-value < 0.05). Younger patients showed and associated high risk of mandibular angle fractures (odds ratio 0.935 (0.893–0.978)) (Table 5). On the other hand, unilateral condyle fractures showed a significant correlation with symphysis, angle, body, and other site fractures in the logistic regression model (*p*-value < 0.05). Patients with unilateral fractures were at low risk (*OR* < 1) of symphysis, angle, body, and other site fractures (Table 6). In the logistic regression analysis model of patients with coronoid fractures, bilateral condyle fractures have a significant correlation with coronoid fractures. Patient with bilateral condyle fractures was at high risk (*OR* > 1) of coronoid fractures (Table 7).

**Table 4:** Logistic regression analysis of patients with symphysis fractures

Factor	*B*	*p*-value	Odds ratio (95% *CI*)
Sex	0.441	0.304	1.554 (0.671–3.599)
Age (year)	−0.019	0.107	0.981 (0.959–1.004)
Angle	−3.399	0.000	0.033 (0.009–0.123)
Body	−4.797	0.000	0.008 (0.001–0.068)
Unilateral condyle	−2.042	0.002	0.130 (0.036–0.474)
Bilateral condyle	0.646	0.524	1.908 (0.261–13.933)
Coronoid process	2.492	0.087	12.083 (0.694–210.260)
Others	−3.489	0.000	0.031 (0.006–0.160)
Cause of trauma (violence)	NA	0.686	NA
Cause of trauma (daily-life activity)	−0.166	0.661	0.847 (0.404–1.777)
Cause of trauma (syncope)	−1.129	0.338	0.323 (0.032–3.253)
Cause of trauma (traffic accidents)	0.373	0.607	1.452 (0.351–6.008)
Medical history	0.267	0.484	1.306 (0.618–2.758)
Smoking	−0.200	0.589	0.819 (0.396–1.691)
Drinking alcohol	0.389	0.247	1.475 (0.763–2.850)

**Table 5:** Logistic regression analysis of patients with angle fractures

Factor	*B*	*p*-value	Odds ratio (95% *CI*)
Sex	−0.364	0.666	0.695 (0.133–3.631)
Age (year)	−0.068	0.003	0.935 (0.893–0.978)
Symphysis	−20.345	0.995	0.000 (0.000)
Body	−21.294	0.994	0.000 (0.000)
Unilateral condyle	−22.297	0.994	0.000 (0.000)
Bilateral condyle	−36.828	0.996	0.000 (0.000)
Coronoid process	0.184	0.910	1.202 (0.050–29.038)
Others	−40.657	0.996	0.000 (0.000)
Cause of trauma (violence)	NA	0.222	NA
Cause of trauma (daily-life activity)	−0.870	0.152	0.419 (0.127–1.379)
Cause of trauma (syncope)	−18.701	0.999	0.000 (0.000)
Cause of trauma (traffic accidents)	−2.046	0.050	0.129 (0.017–0.997)
Medical history	0.557	0.409	1.745 (0.466–6.536)
Smoking	0.561	0.418	1.753 (0.451–6.815)
Drinking alcohol	−0.430	0.488	0.650 (0.193–2.196)

**Table 6:** Logistic regression analysis of patients with unilateral condyle fractures

Factor	*B*	*p*-value	Odds ratio (95% *CI*)
Sex	−0.045	0.939	0.956 (0.301–3.034)
Age (year)	0.016	0.256	1.016 (0.988–1.045)
Symphysis	−1.805	0.018	0.164 (0.037–0.732)
Angle	−5.642	0.000	0.004 (.001–0.021)
Body	−4.390	0.000	0.012 (0.002–0.098)
Bilateral condyle	−22.057	0.998	0.000 (0.000)
Coronoid process	1.539	0.396	4.659 (0.134–162.266)
Others	−3.696	0.000	0.025 (0.004–0.162)
Cause of trauma (violence)	NA	0.610	NA
Cause of trauma (daily-life activity)	0.696	0.180	2.006 (0.724–5.557)
Cause of trauma (syncope)	17.855	0.998	56817712.162 (0.000)
Cause of trauma (traffic accidents)	0.369	0.639	1.446 (0.310–6.744)
Medical history	0.344	0.521	1.410 (0.493–4.032)
Smoking	0.370	0.467	1.448 (0.534–3.928)
Drinking alcohol	−0.300	0.503	0.740 (0.307–1.784)

**Table 7:** Logistic regression analysis of patients with coronoid process fractures

Factor	*B*	*p*-value	Odds ratio (95% *CI*)
Sex	17.703	0.998	48766240.511 (0.000)
Age (year)	−0.033	0.349	0.968 (0.904–1.036)
Symphysis	0.641	0.659	1.898 (0.110–32.612)
Angle	1.314	0.556	3.722 (0.047–297.153)
Body	1.415	0.242	4.118 (0.385–44.058)
Unilateral condyle	1.513	0.250	4.542 (0.344–59.908)
Bilateral condyle	1.948	0.039	7.018 (1.103–44.632)
Others	−15.959	0.998	0.000 (0.000)
Cause of trauma (violence)	NA	0.799	NA
Cause of trauma (daily-life activity)	−0.490	0.716	0.613 (0.044–8.593)
Cause of trauma (syncope)	−19.957	0.999	0.000 (0.000)
Cause of trauma (traffic accidents)	0.901	0.591	2.461 (0.092–65.518)
Medical history	0.787	0.450	2.197 (0.285–16.951)
Smoking	0.018	0.920	1.125 (0.112–11.295)
Drinking alcohol	0.889	0.410	2.432 (0.293–20.169)

## Discussion

The mandible is the only movable bone in the maxillofacial bone, and since the mandible consists of a mechanically weak composition, it is prone to fracture by external forces. So, a thorough understanding of the mechanism of mandibular fractures is important to oral and maxillofacial surgeons. The purpose of this study is to examine a diverse population of mandible fractures to provide a more generalizable assessment of demographic factors, injury mechanisms, and fracture sites across different age groups. So, this study analyzed the correlation of various factors with the distribution of the mandibular fractures. Previous studies showed that the occurrence of mandibular fractures was highly correlated with the age, sex, soft tissue injuries, and pattern and position of the maxillofacial fractures of the patients [3, 7].

In this study, the ratio of males and females in fracture patients was 4.09:1, which was more common in male. This is in conformity with previous studies [8–11]. This is probably due to the higher levels of physical activity that men have than women. And the 20s had the highest frequency of fractures (37.1%) and followed by teens (17.4%). This finding was similar to the results of other previous studies [8, 9, 12, 13]. This is believed to be because people in their 10s and 20s are more active than middle-aged people and are vulnerable to violence [13]. As with previous studies, it was confirmed that there is a more affected age group and a more affected gender group of the mandibular fracture.

The site with the highest frequency of fractures in the maxillofacial region was the symphysis followed by the unilateral condyle and angle. This finding differed from other previous foreign studies [12, 14]. This is because, unlike previous studies, fractures caused by traffic accidents are less frequent in Korean society and are often caused by daily-life activity and violence. Because trauma caused by daily-life activities such as falls and collisions is less severe than trauma caused by a car accident, the scope of trauma was often limited to symphysis. Additionally, the mandible symphysis is located anteriorly in an anatomical position, which makes it easy for external forces to act [6]. The symphysis and unilateral condyle fracture were the most common sites of multiple fractures followed by the symphysis and angle. This finding was similar to other research study [10].

Analysis indicated that daily-life activity such as falling and collision is the major reason for fracture (57.6%). This finding differed from other previous foreign studies [10, 15, 16]. This was very impressive, and it was confirmed that the incidence of mandible fractures caused by violence in Korean society is less frequent than in foreign countries, and the frequency of mandible fractures in sports activities and daily life is high.

According to Table 4, we found that angle, body, and unilateral condyle fractures were associated with symphysis fractures. And we found that symphysis, angle, and body fractures were associated with unilateral condyle fractures (Table 6). From the biomechanical point of view of the symphysis fracture, when an external force is applied to the labial surface of the symphysis, the jaw is flattened, and the lingual cortical plate is simultaneously stretched, resulting in tensile deformation. At this moment when a large external force acts on the parasymphysis on one side, it also acts on the condyle on the opposite side. In patients with unilateral condylar fractures, the condylar process is no longer limited by the articular fossa. The application of an external force widens the mandibular arch and induces tension along the lingual side of the symphysis, making it easy to fracture. So, it leads to the symphysis-unilateral condylar co-fracture [17]. And close relationship has been bilateral condyle fractures and coronoid process fractures. According to Table 7, patient with bilateral condyle fractures was at high risk (*OR* > 1) of coronoid fractures. This was similar to the previous findings [17].

Patients with body and bilateral condyle had little association with other fracture sites. However, in the case of an angle fracture, it was found that there was a significant correlation with age (Table 5). The incidence of angle fracture decreased by 6.5% with increasing age (*OR* 0.935 (0.893–0.978)). Previous studies analyzed the association between mandibular third molar and mandibular angle fractures [18]. The presence of impacted third molars is thought to primarily contribute to the high incidence of angle fractures [19]. So, we hypothesize that this phenomenon occurs because of the presence of impacted third molar. This phenomenon is thought to have occurred because the younger the age, the higher the probability of having impacted third molar [20]. It is considered that additional follow-up studies are needed in this area. In addition, according to previous research, teenagers, especially those between the ages of 16 and 19 years, had a higher prevalence of mandibular angle fractures [13]. This is because angle fractures are often caused by individual assaults [19], and fractures are more frequent by assault in the 10s.

In addition, we thought that falls and traffic accidents may affect the incidence of condyle fracture because external forces are greater than those of beatings. The difference in fracture patterns according to the cause of trauma was analyzed, but it was found that there was no significant correlation. This may be because the cause of the trauma may not have been directly correlated, and the overall patient population was small. Therefore, more follow-up studies are needed. And unlike previous studies, there were no significant results according to medical status, smoking and drinking in all fractures [7]. In addition, age and gender affect the prevalence of mandible fractures, but not on individual mandibular sites.

Interestingly, after the 2020 COVID-19 pandemic, the proportion of violence among the causes of maxillofacial trauma has significantly decreased (35.9%–> 18.3%). In previous studies, the relationship between mandibular fractures and alcohol consumption was proven [21]. This is thought to be because drinking during the night has decreased due to social distancing and lockdown in Korean society, which has led to fewer fights after drinking [22].

## Conclusion

In conclusion, the occurrence of mandibular fracture is related with pattern and position of the maxillofacial fractures of the patients. Although the pattern of fractures has not changed in recent years, some changes have been seen in the etiology factors that affect fractures compared to previous studies. Through this study, it was confirmed that mandible fractures occurred more frequently in males than in females, and they occurred most frequently in their 20s. The site with the highest frequency of fractures in the maxillofacial area was symphysis, which confirmed the difference from previous studies. In addition, the cause of the most fractures was daily-life activity, unlike previous foreign studies. And unlike previous studies, in all mandible fractures, there were no significant results, depending on the medical condition, smoking, and drinking.

Analysis of whether the location of the mandibular fracture affects the fracture of other sites confirmed that several positions have a significant correlation with each other. Through this study, we hope that clinicians can better understand the mechanism of mandibular fracture and reference to the treatment of patients in the future. For example, in patients with bilateral condyle fractures, we must ensure that there are no additional fractures in the coronoid (*OR* 7.018). As such, this study can also provide clinical and research data for the effective management of mandibular fractures.

## Data Availability

Not applicable.

## Abbreviation

OR: Odd ratio
